# Optimization of fucoxanthin extraction obtained from natural by-products from *Undaria pinnatifida* stem using supercritical CO_2_ extraction method

**DOI:** 10.3389/fnut.2022.981176

**Published:** 2022-09-29

**Authors:** Shipeng Yin, Liqiong Niu, Mario Shibata, Yuanfa Liu, Tomoaki Hagiwara

**Affiliations:** ^1^State Key Laboratory of Food Science and Technology, National Engineering Laboratory for Cereal Fermentation Technology, School of Food Science and Technology, Collaborative Innovation Center of Food Safety and Quality Control in Jiangsu Province, National Engineering Research Center for Functional Food, Jiangnan University, Wuxi, China; ^2^Department of Food Science and Technology, Tokyo University of Marine Science and Technology, Tokyo, Japan; ^3^School of Life Sciences, Guangzhou University, Guangzhou, China

**Keywords:** *Undaria pinnatifida* stem, supercritical CO_2_ extraction, optimization, fucoxanthin, inhibitory compounds

## Abstract

In the recent years, edible brown seaweed, *Undaria pinnatifida*, has presented beneficial effects, which may be correlated with this species containing major bioactive compounds, such as carotenoids, fatty acids, and phytosterols. Marine carotenoid fucoxanthin is abundantly present in edible *Undaria pinnatifida* and features strong bioactive activities. The stem of *Undaria pinnatifida* is very hard to gnaw off and cannot be swallowed; therefore, it is usually discarded as waste, making it an environmental issue. Hence, making full use of the waste stem of *Undaria pinnatifida* is an urgent motivation. The present study aims to explore the optimal preparation technology of fucoxanthin from *Undaria pinnatifida* stems using supercritical carbon dioxide methods and provides approaches for the extraction and preparation of bioactive compounds from a waste seaweed part. With the comprehensive optimization conditions applied in this study, the experimental yield of fucoxanthin agreed closely with the predicted value by > 99.3%. The potential of α-amylase and glucoamylase to inhibit bioactive compounds was evaluated. The results demonstrated that the inhibition activity (IC_50_ value) of α-amylase (0.1857 ± 0.0198 μg/ml) and glucoamylase (0.1577 ± 0.0186 μg/ml) varied with extraction conditions due to the different contents of bioactive components in the extract, especially fucoxanthin (22.09 ± 0.69 mg/g extract). Therefore, this study confirmed supercritical fluid extraction technology to be a useful sample preparation method, which can effectively be used to prepare fucoxanthin from waste marine resources. This method can potentially be applied in functional food and related industries.

## Highlights

– Response surface methodology was used to design experiments and optimize the supercritical CO_2_ extraction variables.
– SC-CO_2_ was used to extract fucoxanthin from the waste stem of *Undaria pinnatifida*.
– SC-CO_2_ can be used as an effective method to prepare high-quality fucoxanthin.
– Fucoxanthin prepared by SC-CO_2_ can be used as a potential source of amylase inhibitors.

## Introduction

Seaweed is one of the largest undeveloped and renewable global biomass resources in the world. The global seaweed market is expected to exceed 15 billion USD in value by 2025 according to the latest FAO reports (1–3). Brown seaweeds are edible seaweeds that have been used in the food industry because they are a good source of biomolecules, such as carbohydrates, dietary fiber, proteins, and fats. Recently, many studies revealed that seaweeds contain numerous biologically active phytochemicals, including carotenoids, polysaccharides, polyphenols, sterols, vitamins, minerals, etc. (4–9). *Undaria pinnatifida* is a popular edible brown seaweed, which is widely distributed in Asian countries, including China, Japan, Indonesia, and South Korea (10). In recent years, the aquaculture of *Undaria pinnatifida* increased with the worldwide demand for this species. However, an increase in demand inevitably leads to an increase in seaweed waste streams from industrial processes as well. The stem of *Undaria pinnatifida* is a major waste that is left over from the processing industry, which may potentially lead to wastage of biomass resources and even pose environmental issues (11–16).

Finally, many researchers have reported that *Undaria pinnatifida* has several beneficial effects, including antioxidant, anti-cancerous, anti-hypercholesterolemia, and anti-hypertension properties, which may be correlated with the various bioactive compounds contained by it, such as carotenoids, fucoxanthin, fatty acids, and phytosterols (17–29). Fucoxanthin is a marine carotenoid that is abundant in edible brown algae, contributes over 10% of the estimated total production of natural carotenoids, and shows strong antioxidant, anticancer, antihypertensive, anti-obesity, and anti-inflammatory effects (30–37). As *Undaria pinnatifida* is rich in various bioactive compounds, its unutilized parts may have potential value as raw materials to extract these substances. Until now, several studies have reported the extraction of fucoxanthin and epicatechin from *Undaria pinnatifida*, although most of such works did not specify the parts of *Undaria pinnatifida* that were used.

Currently, organic solvents, such as methanol, ethanol, acetone, and acetonitrile, are used as extraction solvents to obtain useful bioactive compounds, especially fucoxanthin, from *Undaria pinnatifida* (22, 38–40). However, this conventional method may not be the most optimized as residual organic solvents have adverse effects on human health and the environment; they may also damage the functional properties of the extracts (41, 42). In addition, high-temperature processing may result in the degradation of thermally labile compounds upon the separation of solvents from the extracts (43). Therefore, alternative extraction techniques with better selectivity and efficiency have been extensively explored in recent years. Recently, supercritical fluid extraction using carbon dioxide (SC-CO_2_) has come forth as an environment-friendly method for the preparation of bioactive compounds, which also presents a potential alternative to toxic organic solvents. As compared to conventional organic liquid extract, the SC-CO_2_ extract contains fewer polar impurities; therefore, subsequent purification or separation steps are rendered easier (44, 45). For temperature-sensitive compounds, such as fucoxanthin, the SC-CO_2_ method is ideal for extraction because it has a favorable critical temperature and pressure (31.1°C and 7.4 MPa). In addition, the extraction efficiency of fucoxanthin can be significantly improved by adding ethanol as the entrainer (38). The SC-CO_2_ method also presents many advantages for further processing, such as the use of non-toxic, non-flammable, inexpensive, and widely available material with low surface tension, high diffusivity, low viscosity, and suitable density. These aspects enable the CO_2_ to facile penetrate the sample solid biomass matrix and allow rapid mass transfer of desired extracts from the sample matrix to the SC-CO_2_ phase (46, 47). SC-CO_2_ is very sensitive to small changes in experimental parameters, such as temperature, pressure, entrainer, and particle size; therefore, it can be applied to obtain the desired compounds by changing the experimental parameters of selective extractions (38, 48).

The response surface method (RSM), originally proposed by Box and Wilson (49), is characterized by its ability to evaluate the impacts of several process variables and their interaction with response variables, which allows the optimization of the complex extraction process. Therefore, RSM offers many advantages for statistical and mathematical technologies and thus has been successfully used to develop, improve, and optimize processes (50, 51). RSM has been successfully used in modeling and is widely used in the production and optimization of different industrial biotechnology and biochemical processes, especially those related to food systems. Box–Behnken design (BBD) is an RSM model, which can be used to test the relationships between multiple explanatory variables and one or more response variables. It can not only rapidly screen a wide range of conditions but can also indicate the role of each factor (52–54). Recently, RSM was used to model the extraction process and effectively optimize parameters to maximize the recovery of bioactive compounds from plant materials (55, 56).

In this study, the SC-CO_2_ method was investigated for the effective extraction of bioactive compounds from un-utilized *Undaria pinnatifida* stems. RSM was designed by employing a BBD to systemically analyze the effects of extraction parameters, including extraction time, pressure, temperature, particle size, CO_2_ flow, and type of entrainer, on the yields of bioactive compounds, such as fucoxanthin, from the stem. This study will contribute to advancing the knowledge on ideal extraction conditions for obtaining maximal yields of bioactive compounds from the un-utilized stem of seaweed. Our findings can be used as a basis for further studies and provide scientific methods for the extraction and preparation of bioactive compounds from a waste seaweed part.

## Materials and methods

### Materials

The brown seaweed *Undaria pinnatifida* stem was collected from Kesennuma, Miyagi prefecture, Japan, and the harvest time is the middle of April.

High-purity carbon dioxide gas (99%) was supplied by Showa Denko Gas Products Co., Ltd. (Kawasaki, Japan). The amylose B and α-amylase from *Bacillus sp.* and glucoamylase for *Rhizopus sp.* were purchased from Nacalal Tesque, Inc. (Kyoto, Japan). Fucoxanthin standard (≥ 98% purity), sodium dihydrogen phosphate dihydrate (≥ 99% purity), disodium hydrogen phosphate 12-water (99% purity), 1 mol/L sodium hydroxide solution, iodine (99.8% purity), and potassium iodide (99.5% purity) were purchased from Wako Pure Chemical Industries, Ltd. (Osaka, Japan). Milli-Q water was used in these experiments. All reagents used in this study were analytical or high-performance liquid chromatography (HPLC) grade.

### Sample preparation

The *Undaria pinnatifida* stem sample was prepared according to the previous method (13). The lyophilized sample was sieved (100–800 μm) and stored in the freezer (–80°C) before usage.

### Experimental design

The purpose of this experimental design was mainly to identify the optimum conditions from a scale of experiments, which yields the highest amounts of SC-CO_2_ extracts and reveals a large amount of information at the same time (53, 57, 58). A BBD method with three levels and six variables was used for the optimization of extraction variables. The independent variables were extraction time, pressure, temperature, particle size, CO_2_ flow rate, and entrainer. These variables were used to evaluate a combination of extraction variables that can lead to the maximum yields of total flavonoid content and fucoxanthin, as well as enzyme inhibition, in response to the design experiments. The coded and actual values for BBD for the independent variables are shown in Table 1.

**TABLE 1:** Coded levels and actual values of the extract from *Undaria pinnatifida* stem under different conditions (extraction time, pressure, temperature, sample size, CO_2_ flow, and entrainer) in the BBD.

No.	Extraction conditions			No.	Extraction conditions		
	Extraction time (min) (code)	Extraction pressure (psi) (code)	Extraction temperature (°C) (code)		Particle size (μm) (code)	CO_2_ flow (mL/min) (code)	Entrainer (mL) (code)
1	135 (0)	3500 (0)	50 (0)	18	800 (1)	4 (1)	3.125 (0)
2	30 (–1)	1000 (–1)	50 (0)	19	450 (0)	1 (–1)	5 (1)
3	135 (0)	6000 (1)	20 (–1)	20	100 (–1)	2.5 (0)	1.25 (–1)
4	135 (0)	3500 (0)	50 (0)	21	100 (–1)	4 (1)	3.125 (0)
5	240 (1)	6000 (1)	50 (0)	22	450 (0)	2.5 (0)	3.125 (0)
6	135 (0)	3500 (0)	50 (0)	23	100 (–1)	2.5 (0)	5 (1)
7	30 (–1)	3500 (0)	80 (1)	24	450 (0)	2.5 (0)	3.125 (0)
8	135 (0)	1000 (–1)	20 (–1)	25	450 (0)	4 (1)	5 (1)
9	240 (1)	3500 (0)	80 (1)	26	450 (0)	2.5 (0)	3.125 (0)
10	30 (–1)	3500 (0)	20 (–1)	27	100 (–1)	1 (–1)	3.125 (0)
11	30 (–1)	6000 (1)	50 (0)	28	800 (1)	2.5 (0)	5 (1)
12	135 (0)	1000 (–1)	80 (1)	29	800 (1)	2.5 (0)	1.25 (–1)
13	135 (0)	6000 (1)	80 (1)	30	450 (0)	2.5 (0)	3.125 (0)
14	240 (1)	3500 (0)	20 (–1)	31	450 (0)	2.5 (0)	3.125 (0)
15	135 (0)	3500 (0)	50 (0)	32	450 (0)	4 (1)	1.25 (–1)
16	240 (1)	1000 (–1)	50 (0)	33	800 (1)	1 (–1)	3.125 (0)
17	135 (0)	3500 (0)	50 (0)	34	450 (0)	1 (–1)	1.25 (–1)

The predicted response was calculated by a second-order polynomial model, and the response surface analysis is shown in the following equation:


(1) $$Y=b_{0}+\sum_{i=1}^{3}b_{i}\,X_{i}+\sum_{i=1}^{3}b_{i\,i}\,X_{i}+\sum \sum_{i<j=1}^{3}b_{i\,j}\,X_{i}\,X_{j}$$

where Y represents the response variable; *X*_*i*_ and *X*_*j*_ are the independent variables affecting the response; *b*_0_ is the offset term, *b*_*i*_ is the linear effect, *b*_*ii*_ is the squared effect, and *b*_*ij*_ is the interaction effect.

### Supercritical fluid extraction with carbon dioxide

**FIGURE 1:**
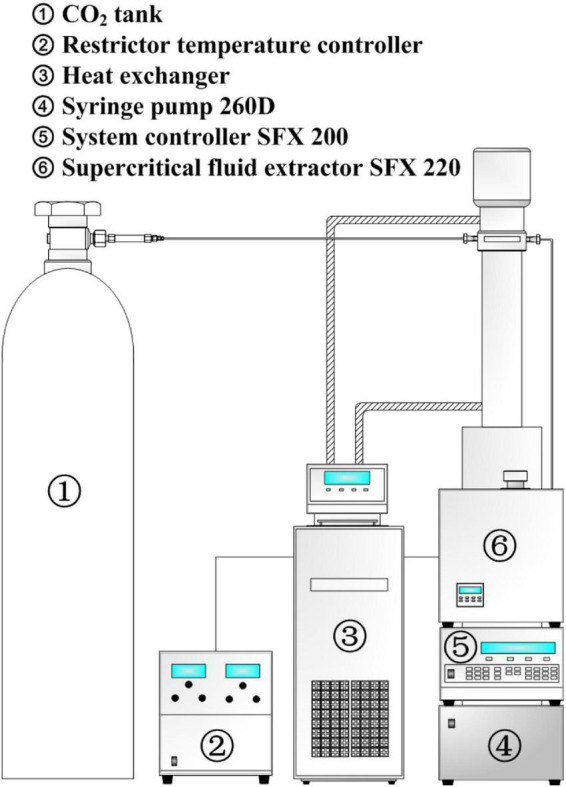
Schematic diagram of supercritical carbon dioxide extraction apparatus.

The independent variables were determined using the above experimental design. A laboratory-scale setup of a supercritical fluid extraction system (Teledyne ISCO Inc., United States) with carbon dioxide (SC-CO_2_) was used (Figure 1) acceding our previous method with modifications. The freeze-dried stem sample (5 g) and different amounts of entrainer ethanol (1.25, 3.125, and 5 ml) were put into a 10-ml extraction cartridge. In total, two pieces of cartridge filters (2 μm; Teledyne ISCO Inc., United States) were placed on the two sides of the extraction cartridge. A syringe pump (260D; Teledyne ISCO Inc., United States) was used to pump the CO_2_ to maintain the pressure and temperature in the extraction chamber. During the extract, different pressure, temperature, and time were used (Table 1). During the static extraction, the chamber outlet valve was closed to prevent CO_2_ from leaking. In the dynamic extraction, the CO_2_ was pumped into the extraction chamber at different flow rates under constant pressure and temperature after opening the valve. A glass tube was then used to trap the eluted CO_2_ with different contents of the entrainer as the crude extract. All the crude extracts were freeze-dried and subjected to the determination of the extraction yield.

### Assay for the determination of total flavonoid content

Total flavonoid content (TFC) in the test sample was measured using previously described methods (13). Absorbance was read at 510 nm at 25°C on a plate reader, and the results were expressed as mg of (+)-catechin equivalents in a gram of total dried crude extract.

### Fucoxanthin analysis by high-performance liquid chromatography

High-performance liquid chromatography analyses were conducted using an HPLC system (SCL-10A; Shimadzu Corp., Kyoto, Japan) equipped with a UV/VIS detector (SPD-20A; Shimadzu Corp.) and a hybrid silica-based ODS column (YMC-Triart C18, 5 × 4.6 mm column size, S-5-μm particle size, 12-nm pore size; YMC Co., Ltd., Kyoto, Japan). The HPLC conditions were similar to those used in earlier studies with minor modifications (13, 43, 59, 60). Methanol–acetonitrile (6:4, v/v) was used as the mobile phase for the analysis of fucoxanthin. All HPLC analyses were conducted at ambient temperature as follows. Solutions of previously dried crude extracts (1 mg/ml in HPLC mobile phase) were filtered using a 0.45-μm membrane filter (Minisart CE 0120; Sartorius Stedim Biotech GmbH, Göttingen, Germany). Approximately, 10 μl of the solutions was injected into the device and eluted at 1 ml/min by the mobile phase as described above. The detection wavelengths were set as 450 nm for fucoxanthin. A standard curve was prepared using an authentic standard of commercial fucoxanthin for the quantification of fucoxanthin in the dried crude extract. The amount of fucoxanthin was quantified from the peak area of the standard curve.

### *In vitro* α-amylase and glucoamylase inhibition assay

The inhibitory activities of the fucoxanthin extract on α-amylase and glucoamylase were assayed using previous methods with modifications (13, 61, 62). In total, four solutions were prepared [labeled 1, 2, 3, and 4 (iodine reagent)]. Solution 1: dried crude extract solution (10–300 μg/ml) in DMSO. Solution 2: amylose solution (0.2% in water). Solution 3: α-amylase and glucoamylase enzyme solution [1 mg/ml in 0.1 M phosphate buffer solvent (pH 7.0)]. Solution 4: iodine reagent solution [5 mM I_2_: 5 mM KI (1:1) dissolved in water]. Initially, the same amount (20 μl) of solutions 1, 2, and 3 was taken in 0.2-ml PCR tubes and incubated in a PCR thermal cycler (Dice mini; Takara Bio Inc., Kusatsu, Japan) to activate the enzymatic reaction at 40°C. As the extract concentrations in solution 1 were 10–300 μg/ml, the final concentration of the extract during this enzymatic reaction process corresponded to 3.33–100 μg/ml, respectively. After 45 min of incubation, 1 M HCl (20 μl) was used to stop the enzymatic reaction. Subsequently, an iodine reagent (100 μl) was used for color development. A microplate reader (Versa Max; Molecular Devices Corp.) was then used at 580 nm to measure absorbance. Enzyme activity was measured as the difference in the absorbance of the enzyme-treated sample compared with that of the untreated sample. The inhibitory activity was expressed as a percentage of difference in absorption between treated and untreated samples. To examine the practicality of the extract as an enzymatic inhibitor, the inhibitory activity of acarbose was also evaluated as the positive control. Acarbose inhibits α- amylase and glucoamylase (63) and thus is used as a medicine to treat diabetes mellitus (64). The final concentration of acarbose during the enzymatic reaction process was set to be the same as the extracts (3.33–100 μg/ml).

IC_50_ values (defined as the inhibitor concentration that inhibits 50% of the enzyme activity of α-amylase and glucoamylase) were determined graphically (percent of inhibition versus log inhibitor concentration) by interpolation from the inhibitions determined on the stem extracts and bioactive compounds (13, 59, 65).

### Statistical analysis

All mean values were analyzed by one-way analysis of variance (ANOVA). All the assays were performed in triplicates and the data were expressed as mean ± standard deviation. Statistical analysis was performed using GraphPad Prism software (version 7.0; GraphPad Software Inc., San Diego, CA, United States), SigmaPlot software (version 12.5; Systat Software Inc., San Jose, CA, United States), or SPSS software for Windows (version 17.0). The *p*-values were determined with corrections by *t*-test or Tukey’s multiple comparisons; *p* < 0.05 was considered statistically significant.

## Results and discussion

### Supercritical fluid extraction with carbon dioxide

Researchers found that seaweed extracts contain a variety of beneficial active substances, such as polyphenols, flavonoids, carotenoids, and so on. As a unique active substance, fucoxanthin is abundant in brown algae. Therefore, the design of SC-CO_2_ extraction technology used in this study will focus on how to obtain fucoxanthin efficiently. Although fucoxanthin is abundant in every part of *Undaria pinnatifida*, previous studies do not report details of its optimal extraction by SC-CO_2_. In addition, only a few researchers have obtained fucoxanthin from the waste part of *Undaria pinnatifida*. Therefore, the extraction conditions of fucoxanthin cannot be inferred from previous research methods. Therefore, new experiments must be designed independently for each variable to explore the best scientific method to obtain fucoxanthin from seaweed. Table 2 provides a brief summary of recent reports on the extraction of fucoxanthin from *Undaria pinnatifida* and the extraction conditions for SC-CO_2_ used in the current research. Important information or conclusions can be derived from Table 2, but these results may be strongly dependent on unforeseen differences in the studies included. There are many experimental factors that can inevitably affect the extraction process, such as variability in extraction parameters and the quality of raw materials.

**TABLE 2:** A brief summary of recent reports on fucoxanthin extraction from different parts of the seaweed by SC-CO_2_ and corresponding extraction conditions.

Sample	Part of sample	Co-solvent	Optimized conditions (Temperature, pressure, time, entrainers, and CO_2_ flow rate)	Fucoxanthin (mg/g DW)	Ref.
*Chara fragilis*	–	Ethanol	40–60°C, 100–300 bar, 2 h, 0–15%,2 mL/min	–	(66)
*Cladophora glomerata*	–	Ethanol	40–60°C, 100–300 bar, 2 h, 0–15%,2 mL/min	–	(66)
*Fucus serratus*	–	Ethanol	30–50°C, 150–300 atm, 1 h, CO_2_: 10 mL/min, ethanol: 0.1 mL/min	0.0715	(67)
*Sargassum horneri*	Blade	Ethanol	45°, 250 bar, 2 h, 710 μm, CO_2_: 27 g/min, ethanol: 1 mL/min	0.77	(60)
*Sargassum japonica*	Blade	Ethanol	45°, 250 bar, 2 h, 710 μm, CO_2_: 27 g/min, ethanol: 1 mL/min	0.41	(60)
*Undaria pinnatifida*	–	Ethanol	40–80°C, 10–40 MPa, 5 h, ethanol: 0.05–0.5 mL/min	0.058	(68)
*Undaria pinnatifida*	Blade	–	25–60°C, 20–40 MPa, 3 h, 60 μm, CO_2_: 1–4 mL/min, ethanol: 2 mL/min	0.682	(48)
*Undaria pinnatifida*	Blade	Ethanol	303–333 K, 80–300 bar, 25 min, 500 μm, CO_2_: 28.17 g/min, ethanol: 2 mL/min	7.53 × 10^–6^	(69)
*Undaria pinnatifida*	Blade	Ethanol	40–70°C, 20–40 MPa, 5 h, 500 μm, CO_2_: 3 g/min, ethanol: 0.05–0.5 mL/min	0.995	(38)
*Undaria pinnatifida*	Stem	Ethanol	40°C, 27.58 MPa, 2 h, 180 μm, CO_2_: 1 mL/min, ethanol: 4.5 mL	0.178	(13)

Therefore, RSM combined with the BBD was selected to investigate six independent variables involved in SC-CO_2_ optimization. According to the characteristics of RSM, we divided the six independent variables into two groups, that is, extraction temperature, time and pressure as one group, and sample particle size, CO_2_ flow rate, and entrainment dose as the other groups. To make the design more scientific reference, these two groups of design models are independent and do not affect each other. The purpose is to obtain the best extraction process through the above two experimental design groups. Table 1 presents a detailed description of the coded and natural values of the selected variables.

**FIGURE 2:**
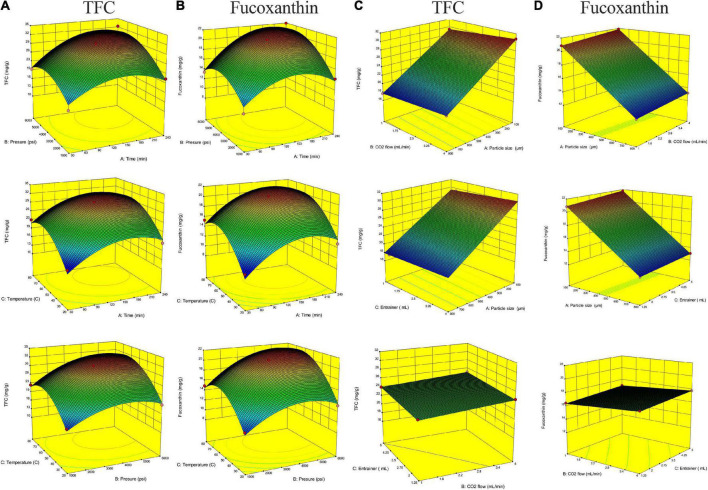
Response surface plots of total flavonoid (TFC) and fucoxanthin content (mg/g) in the extract. **(A,B)** TFC and fucoxanthin versus the particle size (450 μm), CO_2_ flow (2.5 ml/min), and entrainer (1.25 ml). **(C,D)** TFC and fucoxanthin versus the extraction time (135 min), pressure (3500 psi), and temperature (50°C).

**FIGURE 3:**
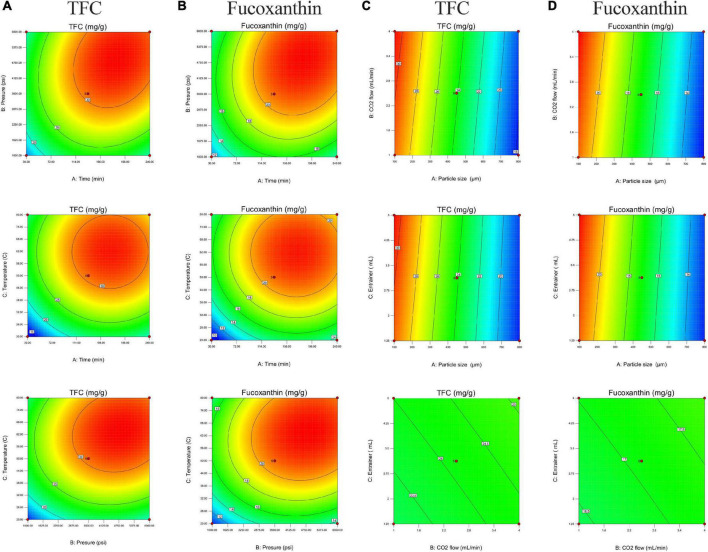
Two-dimensional contour graphs of plots with the optimal points for the total flavonoid (TFC) and fucoxanthin content (mg/g) in the extract. **(A,B)** TFC and fucoxanthin versus the particle size (450 μm), CO_2_ flow (2.5 ml/min), and entrainer (1.25 mL). **(C,D)** TFC and fucoxanthin versus the extraction time (135 min), pressure (3500 psi), and temperature (50°C).

Figure 2 shows the relationship between extraction parameters and bioactive compounds (TFC and fucoxanthin), which were investigated by response surface plots. According to our present study, fucoxanthin is a main monomer active substance in the SC-CO_2_ extract, and TFC (total flavonoids) is a quantitative index of active substance in the SC-CO_2_ extract. This was in agreement with our previous research, although the amount of fucoxanthin was lower than TFC, and the trend of its variability and response surface plot were similar to that of TFC (13, 60). All response plots presented clear peaks with nearly circular contours. Optimum conditions producing maximum values of the responses were attributed to time, pressure, and temperature in the design space. The contour graphs showed incomplete concentrically closed curves, whose centers represented optimum conditions (Figure 3). Interestingly, data showed an increase in TFC and fucoxanthin with an increase in time, pressure, and temperature during the preliminary stage. The highest amounts of TFC and fucoxanthin were found during the mid-term with time, pressure, and temperature continuously increasing. During the later stages, a decrease in TFC and fucoxanthin was observed with a progressive increase in time, pressure, and temperature. This trend maintained itself until the end. In an analysis of the results from the above prediction model experiment, the highest TFC (32.15 ± 0.11 mg/g) and fucoxanthin (21.93 ± 0.20 mg/g) yields were provided by Experiment 5 (time = 240 min, pressure = 6,000 psi, and temperature = 50°C) among the first 17 experiments (Table 3).

**TABLE 3:** Box–Behnken design of factors with total flavonoid content (TFC) and fucoxanthin based on experimental and predicted values under general particle size (450 μm), CO_2_ flow (2.5 ml/min), and entrainer (1.25 ml).

No.	Yield (%)		Analytical results (mg/g extract)			
			TFC^1^		Fucoxanthin	
	Experimental	Predicted	Experimental	Predicted	Experimental	Predicted
1	1.24 ± 0.10	1.24	30.48 ± 0.09	30.48	20.78 ± 0.19	20.78
2	0.62 ± 0.02	0.70	15.31 ± 0.01	17.09	10.39 ± 0.02	11.62
3	0.78 ± 0.06	0.81	19.39 ± 0.03	19.99	13.17 ± 0.08	13.59
4	1.24 ± 0.09	1.24	30.50 ± 0.09	30.48	20.79 ± 0.17	20.78
5	1.31 ± 0.11	1.24	32.15 ± 0.11	30.37	21.93 ± 0.20	20.71
6	1.24 ± 0.10	1.24	30.47 ± 0.09	30.48	20.77 ± 0.18	20.78
7	0.91 ± 0.07	0.86	22.51 ± 0.04	21.33	15.31 ± 0.11	14.50
8	0.65 ± 0.02	0.62	16.04 ± 0.01	15.41	10.89 ± 0.03	10.46
9	1.09 ± 0.07	1.14	26.89 ± 0.07	28.04	18.31 ± 0.15	19.10
10	0.59 ± 0.01	0.54	14.58 ± 0.01	13.43	9.89 ± 0.02	9.10
11	0.80 ± 0.07	0.82	19.79 ± 0.03	20.34	13.45 ± 0.07	13.83
12	0.89 ± 0.07	0.86	21.94 ± 0.04	21.34	14.92 ± 0.09	14.50
13	1.19 ± 0.09	1.22	29.37 ± 0.08	30.00	20.02 ± 0.18	20.45
14	0.76 ± 0.05	0.81	18.83 ± 0.03	20.01	12.79 ± 0.05	13.60
15	1.24 ± 0.09	1.24	30.48 ± 0.09	30.48	20.78 ± 0.18	20.78
16	0.84 ± 0.07	0.82	20.91 ± 0.04	20.36	14.21 ± 0.11	13.84
17	1.24 ± 0.10	1.24	30.47 ± 0.10	30.48	20.77 ± 0.19	20.78
18	0.77 ± 0.05	0.78	18.64 ± 0.03	18.66	13.18 ± 0.01	13.19
19	1.00 ± 0.07	1.00	24.02 ± 0.05	24.00	17.02 ± 0.06	17.02
20	1.23 ± 0.09	1.24	29.38 ± 0.07	29.48	20.86 ± 0.09	20.92
21	1.29 ± 0.09	1.29	30.78 ± 0.07	30.74	21.86 ± 0.10	21.84
22	1.01 ± 0.08	1.01	24.12 ± 0.05	24.14	17.10 ± 0.06	17.11
23	1.28 ± 0.10	1.28	30.67 ± 0.07	30.67	21.79 ± 0.09	21.78
24	1.01 ± 0.07	1.01	24.12 ± 0.05	24.14	17.10 ± 0.06	17.11
25	1.05 ± 0.08	1.05	25.08 ± 0.05	25.09	17.78 ± 0.05	17.79
26	1.01 ± 0.07	1.01	24.12 ± 0.05	24.14	17.10 ± 0.06	17.11
27	1.23 ± 0.09	1.24	29.41 ± 0.06	29.40	20.88 ± 0.08	20.87
28	0.77 ± 0.04	0.77	18.48 ± 0.03	18.43	13.07 ± 0.01	13.03
29	0.74 ± 0.04	0.74	17.92 ± 0.02	17.97	12.68 ± 0.01	12.71
30	1.01 ± 0.07	1.01	24.12 ± 0.05	24.14	17.10 ± 0.05	17.11
31	1.01 ± 0.07	1.01	24.12 ± 0.04	24.14	17.10 ± 0.06	17.11
32	1.02 ± 0.07	1.02	24.39 ± 0.05	24.31	17.29 ± 0.06	17.23
33	0.73 ± 0.04	0.73	17.69 ± 0.02	17.74	12.51 ± 0.01	12.54
34	0.97 ± 0.06	0.97	23.25 ± 0.04	23.14	16.47 ± 0.03	16.40

^1^TFC as catechin equivalents. Values are expressed as means ± SD (*n* ≥ 3).

In Experiments 18–34, the TFC and fucoxanthin exhibited high contents under fixed times, pressure, and temperature.

The effect of particle size on extraction rate was significant, which was indicated by the response surface and contour diagrams shown in Figures 1, 2. The response diagram, involving the change in particle size as the independent variable, shows a clear trend; here, the shape was similar to a landslide. Optimal conditions for maximal responses were attributed to the particle size in the design space, where the maximum response tended to exhibit a trend opposing the particle size. This trend was maintained throughout the process. However, the effect of CO_2_ flow and entrainer was not significant. The highest amounts of bioactive compounds were observed on the smallest particles (100 μm), regardless of CO_2_ flow rate and entrainer amount. These amounts were higher as compared to the amounts of bioactive compounds on larger particles of 450 and 800 μm diameters. Therefore, the best yields of TFC (30.78 ± 0.07 mg/g) and fucoxanthin (21.86 ± 0.10 mg/g) were obtained under Experiment 21 (particle size = 100 μm, CO_2_ flow rate = of 4 mL/min, and entrainer = 3.125 ml) (Table 3).

New experiments were designed to explore the effects of extraction temperature, pressure, and time on the yield of fucoxanthin in the next study and to verify the aforementioned conclusions.

### Influence of independent variables on investigated responses

Over the years, there have been many successful cases of industrial production of raw materials using SC-CO_2_ (70). However, the extractions did not focus only on oil, but also placed value on the by-products or the use of co-extraction using SC-CO_2_ to enhance the properties of the final products (6, 70). In this research, fucoxanthin, as the main substance in the extract, was the key natural product. Previous studies have shown that supercritical extraction of high molecular weight natural products (such as oils, pigments, and antioxidants) usually requires high pressure (above 270 bar) (71, 72). Therefore, some targeted experiments have been carried out in this research to verify the above conclusions.

**FIGURE 4:**
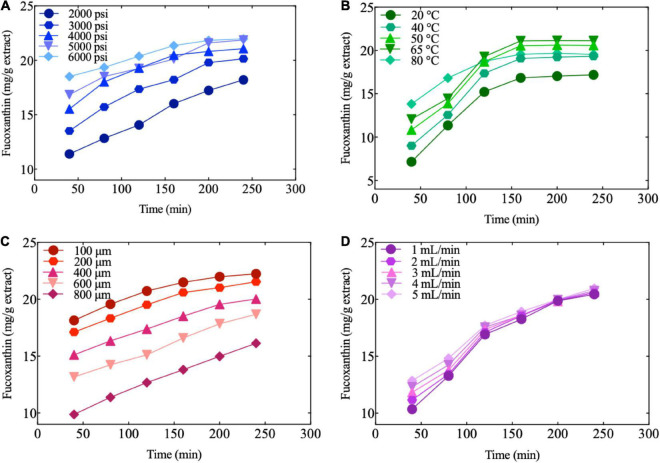
Fucoxanthin extraction by different conditions. **(A)** Effect of pressure on the amount of fucoxanthin produced as a function of time at constant extraction temperature, sample size, CO_2_ flow, and entrainer (60°C, 450 μm, 2.5 ml/min, and 1.25 ml); **(B)** Effect of temperature on the amount of fucoxanthin produced as a function of time at constant extraction pressure, sample size, CO_2_ flow, and entrainer (4000 psi, 450 μm, 2.5 ml/min, and 1.25 ml); **(C)** Effect of sample size on the amount of fucoxanthin produced as a function of time at constant extraction pressure, temperature, CO_2_ flow, and entrainer (3500 psi, 50°C, 2.5 ml/min, and 1.25 ml); **(D)** Effect of CO_2_ flow on the amount of fucoxanthin produced as a function of time at constant extraction pressure, temperature, sample size, and entrainer (3500 psi, 50°C, 400 μm, and 1.25 ml).

The appearance of the extracts was liquid-like with an oily texture, which was enhanced with an increase in pressure at a constant temperature. Figure 4A shows that higher pressure had a significant influence on the extraction efficiency of fucoxanthin at constant temperature and time. This trend could be attributed to a direct increase in the density due to SC-CO_2_. Meanwhile, the increased pressure could strengthen the physical intermolecular interactions, increasing the solvent power. Amosova et al. (73) optimized the process parameters of SC-CO_2_ extraction and reported that the extraction rate of carotene was approximately 35% at higher pressure (350 atm). According to Machmudah et al. (38) although the amounts of the total extract and extracted astaxanthin were not significantly affected by increasing pressure at lower levels, a dramatic increase was observed at higher pressures. The dependency on pressure is expected to increase CO_2_ density at higher pressure, hence the solvent’s capability to dissolve substances.

As can be seen from Figure 4B, at the same extraction time and pressure, the temperature had the most significant influence on fucoxanthin than pressure and time. Initially, the amount of fucoxanthin extracted increased significantly with the increase in temperature. Subsequently, the amount of fucoxanthin did not increase significantly and maintained a uniform trend until the end. These results indicated that the solute vapor pressure increased with an increase in temperature and hence improved the extraction efficiency of fucoxanthin. In addition, it is possible that the increase in temperature causes the cell wall to decompose, thereby increasing the content of fucoxanthin and extractable compounds (38). In addition, Figure 4B also shows that the extraction rate of fucoxanthin was enhanced more by an increase in temperature due to the more obvious effect of temperature on solubility than the increase in pressure. Fucoxanthin is a kind of carotenoid, which is generally characterized as heat-sensitive; therefore, fucoxanthin may degrade at high temperatures (74). In our research, the amount of fucoxanthin extracted at 80°C was slightly lower than that at 50°C. This is in line with previous research, which also found the best temperature for extraction of carotenoids from pumpkins by SC-CO to be 50–70°C (75).

Figures 4C,D show the influence of the sample particle sizes and CO_2_ flow rate on fucoxanthin extraction at constant extraction time, pressure, and temperature. The sample particle size had a significant effect on the extraction efficiency of fucoxanthin, and the particle size was negatively correlated with the extraction rate. The extraction of fucoxanthin significantly increased at 100 μm, which was attributable to the increase in the contact surface of supercritical solvent upon grinding; fucoxanthin was released readily from the broken cells, resulting in better extraction of the soluble bioactive components. It was also indicated by previous studies that particle size affects the mass transfer resistance and controls the diffusion of CO_2_ into the soluble matrix. The use of smaller particle sizes could achieve better efficiencies of extraction, which not only increased the surface of mass transfer, but also increased the number of soluble fractions on the surface (76, 77). These results agreed with the findings of José and Del-Valle (78), Nagy et al. (79), and Asep et al. (80), who similarly reported extraction yield to significantly increase by a decrease in particle size.

By setting particle size at a constant level (400 μm), it could be seen that regardless of whether CO_2_ flow and entrainer were at low or high levels, the effect on the number of bioactive compounds was minimal (Figure 4D).

### Optimization of extraction parameters and validation of the model

The applicability of the model equation to predict the optimal response value was tested using slightly modified optimal parameters. We compared the maximum predicted yield and experimental yield of bioactive compounds. Additional experiments using the predicted best parameters for the extraction of bioactive compounds were as follows: extraction time, pressure, temperature, particle size, CO_2_ flow rate, and entrainer were set at 240 min, 50°C, 6000 psi, 100 μm, 4 ml/min, and 3.125 ml, respectively. To ensure that the predicted results did not deviate from the actual values, the following modified parameters were experimentally reviewed: extraction time of 230 min, temperature of 53°C, pressure of 5900 psi, and entrainer of 3.0 ml. The values obtained from actual experiments (Table 4) agreed with the predicted values significantly (*p* > 0.05), demonstrating the effectiveness of the RSM model. A good correlation between these results confirmed that the RSM was sufficient to reflect the expected optimization. The results showed that the experimental values were in good agreement with the predicted values. The model from BBD accurately and reliably predicted the bioactive compounds, especially fucoxanthin, from steam by SC-CO_2_ extraction.

**TABLE 4:** Experimental and predicted values of responses by SC-CO_2_ extraction^1^.

	Experimental (mg/g)	Predicted (mg/g)
TFC	31.76 ± 0.05*	30.12
Fucoxanthin	20.42 ± 0.04*	20.51

^1^Extraction time = 230 min, temperature = 48°C, pressure = 5800 psi, entrainer = 3.0 mL, particle size = 100 μm, and CO_2_ flow rate = 4 ml/min. *Significant at 0.05. Values are expressed as means ± SD (*n* ≥ 3).

There are mainly two kinds of raw materials for obtaining fucoxanthin. One is brown algae, such as *Saccharina japonica*, *Undaria pinnatifida*, *Sargassum fusiforme*, and *Eisenia bicyclis*, etc. Research shows that each gram of macroalgae contains 0.1–1.0 mg fucoxanthin (81–83). However, brown seaweed can not meet the global demand for fucoxanthin due to the long growth cycle, limited production, and marine environmental pollution (84, 85). The other is microalgae represented by diatoms. It is regarded as a more promising organism to produce fucoxanthin. The current research shows that the fucoxanthin output of diatoms can reach 26.6 mg/g DW, much higher than brown algae (84). Researchers have made significant progress in fucoxanthin biosynthesis and microalgae physiology in recent years. However, there are still some problems in the production of fucoxanthin by microalgae, such as the unclear biosynthesis mechanism is not comprehensive enough, the microalgae cultivation process needs to be continuously optimized and the innovation of microalgae fucoxanthin extraction technology (85).

The brown algae used in this study is *Undaria pinnatifida*. It is reported that its maximum yield is 7.53 mg/g DW, far lower than microalgae (69). However, the material used in this study is the *Undaria pinnatifida* waste steam. The complete calculation results are 0.282 mg/g DW, and the yield is not very high. As we all know, *Undaria pinnatifida* is a popular and widely planted algae, which will produce a large amount of waste when receiving goods every year. Suppose it can be effectively developed and utilized. In that case, it can not only solve the waste of resources and environmental pollution but also create a certain value, which is of great significance.

### Enzyme inhibition assay

Optimum conditions for extraction of active substances were obtained by RSM, and the applicability of the model was verified. After that, the amylase inhibitory activity of the extracted fucoxanthin was briefly evaluated.

**FIGURE 5:**
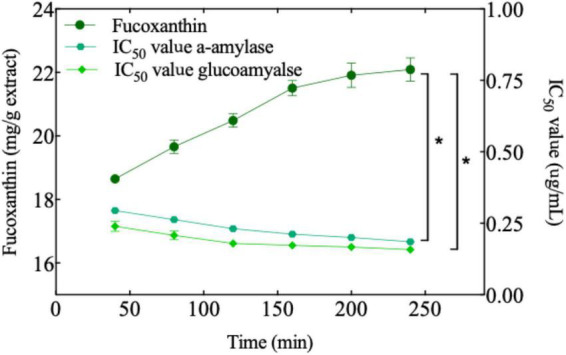
The amount of fucoxanthin produced as a function of time at constant extraction pressure, temperature, sample size, CO_2_ flow, and entrainer (6000 psi, 65°C, 450 μm, 2.5 ml/min, and 1.25 ml). The IC_50_ value of several experiments against two enzymes (α-amylase and glucoamylase). A significant difference (*t*-test, *p* < 0.05) is indicated with an asterisk.

In previous studies, flavonoid compounds have been reported to have amylase inhibition, which was related to the flavonoid content (86–89). In our study, temperature, pressure, and sample size had a significant impact on the extraction rate of bioactive compounds. Therefore, we designed an experiment to extract the active substances at a constant temperature, pressure, and sample particle size, followed by an evaluation of their amylase inhibition. As can be seen from Figure 5, in the beginning, the amount of fucoxanthin increased significantly up to 160 min, while having a significant effect on amylase inhibition. After 160 min, amylase inhibition by the extract increased continuously, but it did not change with the change in fucoxanthin content. It is probable that there were other active substances in the extract in addition to fucoxanthin. Although glucoamylase inhibition exhibited the same trend as α-amylase, the difference was that the inhibitory ability of fucoxanthin for glucoamylase was a little higher than that for α-amylase. This could be because the amount of fucoxanthin extracted as the main active substance was high, and amylase inhibition could be more dependent on some specific structures. The researcher proposed that flavonoids have a significant effect on α-amylase, and glucoamylase has obvious inhibitory activity due to the particular structure of flavonoids (87, 90–92). The OH group of flavonoids can be combined with the carboxylic acid groups (Glu233 and Asp197) in the active site of α-amylase form hydrogen bonds, which in turn affect the activity of amylase (91). Therefore, the two OH groups in fucoxanthin may play a similar role in the binding process with amylase. In addition, the long hydrocarbon chain of fucoxanthin containing conjugated double bonds may have hydrophobic interaction with the amino acid residues in amylase, further enhancing the biological activity of fucoxanthin. Through molecular simulation, researchers found that the additional hydrogen bond in fucoxanthin may play a role in stabilizing the open form of protein tyrosine phosphatase 1B (PTP1B), further strengthening the enzyme inhibition ability of fucoxanthin (90). Although the above research does not directly explain that fucoxanthin has a typological effect on amylase, we may be inspired to conduct more accurate experiments. As has been reported previously, there is a correlation between flavonoid content and amylase inhibition. However, although the yield of fucoxanthin did not increase significantly with a continuous increase in extraction time, amylase inhibition by the extract increased continuously. It could be due to the extraction of other bioactive substances, which affected the inhibition of amylase. Many previous studies have indicated that the content of bioactive compounds is an important parameter for the inhibition of enzyme activity (93, 94).

The correlation between fucoxanthin content and enzyme inhibition ability was analyzed. A significant correlation was found between the two parameters. However, it must be noted that the bioactive extracts are not single compounds, and synergistic or antagonistic effects may originate from some specific compounds or a combination of specific compounds.

### Challenges in applying proposed extraction technology

Traditional methods, such as solid or liquid extraction, are generally used to obtain natural bioactive substances from the waste parts of plants. Owing to the application of natural bioactive compounds in the food and pharmaceutical industries, the market demand for these compounds is growing rapidly. The disadvantages of traditional methods are as follows: (1) new regulations on the use, residue production, and contamination control of toxic chemicals; (2) products from traditional processing technology cannot meet the market demand for higher quality products; and (3) consumers’ concerns about the safety of final products. Therefore, it is necessary to develop extraction technologies that can provide high-quality extracts. In this study, we have proven that supercritical fluid extraction is a clean and efficient extraction technology for bioactive compounds from seaweed. Although this technology has some shortcomings, such as the requirement of a huge investment in the early stage of setup, its advantages are obvious in the long term. With the rapid development of commercial applications of supercritical technology, more research studies are focusing on the real application of this technology, rather than remaining limited to laboratory research.

Like fucoxanthin, which is the focus of this research, it has great commercial potential. At present, the price of high-purity fucoxanthin (more than 90%) raw materials in the market is as high as 60,000 US dollars a kilogram (88). In addition, when fucoxanthin has been applied to practical healthcare products, although the content of effective components is not very high, it has attracted much attention due to its special efficacy, and its product price can reach more than 5,000 US dollars a kilogram. Representative products are Fucoxanthin EX and FUCOXAN’NOL D products from the Japanese market, FucoThin Softgels products from the Garden of life, and Brown seaweed products from Horbaach (95–98).

## Conclusion

In the present study, a response surface methodology with the BBD coupled with a numerical optimization technique was applied to optimize and study the interactive effects of different process variables on the SC-CO_2_ extraction of an important bioactive functional component, fucoxanthin, from unused *Undaria pinnatifida* stem. The results demonstrated that extraction temperature and pressure, particularly, particle size, are the key parameters controlling the extraction of fucoxanthin, whereas the impact of other factors is limited. In terms of bioactive compounds, high yields of fucoxanthin were found in *Undaria pinnatifida* stem. Hence, this study’s experimental results highlight several key advantages of SC-CO_2_: (1) use of less solvent/CO_2_ consumption; (2) less residual solvent in the extract, even in the case of co-solvent; (3) the rationality of the SC-CO_2_ process, such as no excessive separation or purification steps, high purity of the bioactive product (fucoxanthin); and (4) controllability of the temperature in the extraction process, allowing adaptation of the production to the thermally sensitive fucoxanthin. Therefore, SC-CO_2_ extraction of fucoxanthin from seaweed stem may be used to develop dietary supplemental products for the management of different biomolecules, such as the digestion of amylose in dietary starches, and to improve the potential for their effective utilization. Therefore, this study may become the basis for future research on a large-scale extraction system in food and pharmaceutical industries and can accelerate the sustainable utilization of commodity waste from seaweed.

## Data availability statement

The original contributions presented in this study are included in the article/Supplementary material, further inquiries can be directed to the corresponding author.

## Author contributions

SY: conceptualization, investigation, writing—review and editing the manuscript. LN, MS, YL, and TH: review the manuscript. All authors have read and agreed to the published version of the manuscript.
