# Supplementary material for: Optimization of fucoxanthin extraction obtained from natural by-products from Undaria pinnatifida stem using supercritical CO2 extraction method
Source: Front Nutr. 2022 Sep 29;9:981176. doi: 10.3389/fnut.2022.981176 (PMC9558218; doi:10.3389/fnut.2022.981176)
Supplement: Supplementary file 1 [file Data_Sheet_1.PDF]

Supplementary data of

Optimization of fucoxanthin extraction obtained from natural by-products from *Undaria pinnatifida* stem using supercritical CO_2 extraction method

*Shipeng Yin[1,2], Liqiong Niu[3], Mario Shibata[2], Yuanfa Liu[1], Tomoaki Hagiwara[2 *]*

[1] State Key Laboratory of Food Science and Technology, School of Food Science and Technology, Collaborative Innovation Center of Food Safety and Quality Control in Jiangsu Province, National Engineering Reacher Center for Functional Food, National Engineering Laboratory for Cereal Fermentation Technology, Jiangnan University, No. 1800 Lihu Road, Binhu District, Wuxi, 214122, China

[2] Department of Food Science and Technology, Tokyo University of Marine Science and Technology, Konan 4-5-7, Minato-ku, Tokyo, 108-8477, Japan

[3] School of Life Sciences, Guangzhou University, Guangzhou, 510006, China

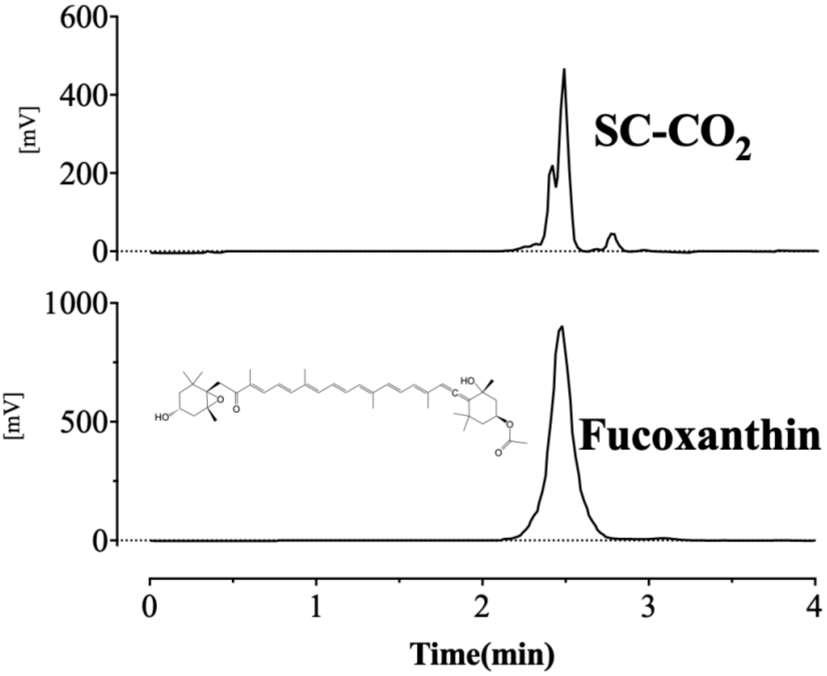

Figure: Typical HPLC chromatograms of the different comparison with fucoxanthin standard. SC-CO2: Extraction time = 150 min, temperature = 40°C, pressure = 4000 psi, entrainer = 1.0 mL, particle size = 450 μm, and CO_2 flow rate = 1 mL/min
